# Functional Advantages of Conserved Intrinsic Disorder in RNA-Binding Proteins

**DOI:** 10.1371/journal.pone.0139731

**Published:** 2015-10-06

**Authors:** Mihaly Varadi, Fruzsina Zsolyomi, Mainak Guharoy, Peter Tompa

**Affiliations:** 1 Structural Biology Research Center (SBRC), Flemish Institute of Biotechnology (VIB), Brussels, Belgium; 2 Structural Biology Brussel (SBB), Vrije Universiteit Brussel (VUB), Brussels, Belgium; 3 Institute of Enzymology, Research Centre for Natural Sciences, Hungarian Academy of Sciences, Budapest, Hungary; Weizmann Institute of Science, ISRAEL

## Abstract

Proteins form large macromolecular assemblies with RNA that govern essential molecular processes. RNA-binding proteins have often been associated with conformational flexibility, yet the extent and functional implications of their intrinsic disorder have never been fully assessed. Here, through large-scale analysis of comprehensive protein sequence and structure datasets we demonstrate the prevalence of intrinsic structural disorder in RNA-binding proteins and domains. We addressed their functionality through a quantitative description of the evolutionary conservation of disordered segments involved in binding, and investigated the structural implications of flexibility in terms of conformational stability and interface formation. We conclude that the functional role of intrinsically disordered protein segments in RNA-binding is two-fold: first, these regions establish extended, conserved electrostatic interfaces with RNAs via induced fit. Second, conformational flexibility enables them to target different RNA partners, providing multi-functionality, while also ensuring specificity. These findings emphasize the functional importance of intrinsically disordered regions in RNA-binding proteins.

## Introduction

The interactions between RNA and protein molecules are essential for molecular processes both in cellular organisms, where they govern the assembly of the protein synthesizing macromolecular machineries, such as the ribosome and spliceosome[1], and in viruses, where they envelope the (RNA) genetic material by capsid formation[2], or enhance the efficiency of viral transcription [3]. Due to their central role, protein-RNA complexes have been extensively studied and it soon became apparent that RNA-binding proteins are enriched in intrinsic structural disorder [4]. As a consequence of conformational flexibility, these proteins often go through binding-induced folding[5]. Such disorder-to-order transitions appear ubiquitously, and in RNA-protein interactions conformational changes can occur either in the structure of the protein, the RNA partner, or both [6]. However, disorder-to-order transitions entail special energetic consequences on the interaction, because a fraction of the available binding enthalpy needs to compensate for the entropic cost of the conformational changes [5]. Therefore, even though structural disorder is prevalent in RNA-binding proteins, the advantages that would emanate from their prevalent bonding mode, remain elusive. Further, conformational flexibility could be the by-product of the optimization of electrostatic interactions, since RNA-binding proteins have to be enriched in positively charged amino acids in order to establish favourable electrostatic interactions with the highly negatively charged RNA surfaces [7, 8]. Since charged residues are disorder promoting, they may destabilize the protein chain at the same time.

Understanding the role of structural disorder in protein-RNA interactions would be instructive in explaining the prevalence of conformational flexibility in general, and could provide valuable insights regarding complex assembly and the regulation of the molecular processes these complexes are involved in. Is disorder a by-product of binding optimization in terms of electrostatic interactions, or is there more to the prevalence of conformational flexibility, than meets the eye?

Here, we present a comprehensive computational analysis based on both sequence and structural data in an attempt to elucidate if protein function correlates with intrinsic disorder in RNA binding proteins. We demonstrate the extent of structural disorder in the known RNA-binding proteins in an evolutionary context, investigate the consequences of conformational flexibility and test a number of plausible explanations for the enrichment of intrinsic disorder in this family of proteins.

## Materials and Methods

### Data Retrieval and Processing

The analyses were based on three data sets from the following sources: The Pfam27.0 database [9], the UniProt/SwissProt[10] database and the Protein Data Bank [11].

The Pfam dataset contained the full length protein sequences of 344 DNA-binding and 140 RNA-binding domain families. The sequences were filtered against redundancy using CD-HIT [12], and the domains were extracted using Pfam scan [9] yielding 406,736 unique DNA-binding and 210,962 unique RNA-binding protein domain sequences. The complete Pfam-A dataset (10,626,097 domain sequences), excluding the RNA- and DNA-binding domains, was used as a reference.

From UniProt/SwissProt we retrieved 43,111 unique DNA-binding protein sequences (GO:0003677) and 66,386 unique RNA-binding protein sequences (GO:0003723) based on GO-terms. The full SwissProt dataset excluding RNA- and DNA-binding proteins was composed of 542,782 protein sequences and served as a reference.

Finally, we retrieved 2877 unique DNA-protein complexes and 1605 unique RNA-protein complexes, along with 4278 protein-protein complexes. 1420 unique DNA-binding, and 1293 unique RNA-binding protein sequences were extracted from the complexes, and compared to the entire PDB dataset consisting of 57,041 unique protein sequences, excluding RNA- and DNA-binding proteins. The sequences in FASTA format for each dataset can be downloaded from http://pedb.vib.be/discons/data.tar.gz or from Dryad (doi:10.5061/dryad.33vn1).

### Disorder Predictions and Conservation Analysis

Previously, we have developed DisCons [13](available at http://pedb.vib.be/discons), a novel and freely accessible tool that serves two purposes: first, it provides a position-specific conservation score of protein disorder in the context of a multiple sequence alignment (MSA). Second, it classifies each position by combining the conservation scores of the sequence and of the structural disorder, following the protocol of *Bellay et al*.[14].

Briefly, the calculation procedure is the following: during the initial step, DisCons performs a PSI-BLAST search against a specified collection of protein sequences; we used the UniProt/SwissProt database for this purpose in our analysis. Hits that satisfy the pre-set thresholds are then aligned using MAFFT [15] and the MSA serves as an input for the next steps of the DisCons pipeline. Position-specific sequence conservation scores (SCS) are calculated based on the MSA by the scoring method of Capra et al.[16]with Jensen-Shannon divergence and a window of size 3. Scores range from 0 (diverse) to 9 (strongly conserved). Next, disorder scores are calculated using IUPred [17] and are mapped onto the MSA. Position-specific disorder conservation scores (DCS) are determined by calculating the fraction of positions for which the disorder score is 0.5 or greater, with scores also ranging from 0 (non-conserved) to 9 (highly conserved).

Finally, for those MSA positions where the fraction of gaps across the aligned sequences is less than 30%, the sequence- and disorder conservation scores are combined, and positions are classified as having i.) 'Constrained' disorder, if both scores are 0.5 or greater; ii.) 'Flexible' disorder, if the sequence conservation score is lower than 0.5, but the disorder conservation score is 0.5 or greater; iii.) 'Non-conserved' disorder, if the disorder conservation score is lower than 0.5, but higher than 0; and finally, iv.) 'Structured', if the disorder conservation score is 0, indicating the complete lack of disorder at the given position.

### Identifying Secondary Structural Elements and Interface Residues

Secondary structure assignments for protein chains were obtained by the DSSP algorithm[18]. The secondary structure types considered were: alpha- and 3_10_-helix, beta-strand, turn (with or without hydrogen bonding), and unclassified. The two helical types and strands were considered as ‘regular’ secondary structures, whereas turns and unclassified types were labelled ‘non-regular’ secondary structures, according to the previously described protocol of *Guharoy et al*.[19].

Protein residues in direct contact with RNA were identified by pair-wise distance calculations between protein and RNA chains: contacts that consisted of 5 or more atom pairs within 5Å were recorded interface contacts, effectively identifying 19128 protein-RNA interfaces. The residues of these interfaces were investigated in terms of sequence- and disorder conservation using the DisCons pipeline described in the previous section.

### Conformational Stability and Surface Accessibility Plots

Conformational stability of the protein chains in 1605 RNA-protein, 2877 DNA-protein and 4278 protein-protein complexes was calculated using FoldX[20]. The bound conformations of the proteins were separated from the complexes, and the calculations were performed on every single chain.

We plotted the accessible surface area as a function of the accessible interface area, normalized by the number of residues, as suggested by Nussinov[4, 21], where structured proteins are located on the lower left side of the plot, below the threshold line of 80, while (disordered) proteins flexible in their free form are found in the upper right side [4, 21]. In order to create the plots, the whole surface area, the chain surface area and the complement surface area have been calculated for each complex using in-house Python scripts, and the interface areas were defined as the whole complex area subtracted from the sum of the chain surface area and the complement surface area. This value was divided by two to take into account only one side of the interface. The calculations involved the usage of the PDBParser module of the Bio.PDB package [22] and the PyMol package to calculate the areas via The PyMol Molecular Graphics System, Version 1.5.0.4 Schrödinger, LLC. We used the parameter “dot solvent” set to “on” so that the solvent accessible surface area was taken into account. The accessible surface area and the interface area were both normalized to the number of residues.

### Investigating the Number of Interaction Partners in RNA-Protein Complexes

The number of unique interaction partners for RNA-binding protein chains was calculated using in-house Python scripts that used the PDBParser module of Bio.PDB [22]. For PDB entries with multiple models, only the first model was taken into consideration. Interacting residues were defined as residues having at least one atom (each) with a maximum distance of 5 Å from each other. We considered two chains as interaction partners if they had at least 5 interacting residues.

### Statistical Analyses

Data processing, exploratory data analyses and statistical tests were performed in the R statistical programming environment using RStudio. Welch t-tests were performed where the distributions were not Gaussian and we could not assume equal variances. Kolmogorov-Smirnov tests were performed in cases where the only valid assumption regarding the variables was their continuity. A p-value of 2.2e-16 is the lowest precision point allowed in R, and implies extremely high significance.

## Results

### Large Scale Investigation of Structural Disorder Reveals Ubiquitous Enrichment of Conserved Flexibility in RNA-Binding Proteins

The prevalence of intrinsically disordered regions in RNA-binding proteins is well documented, yet the reasons behind this phenomenon and its consequences are not fully explored[5, 23]. In order to comparatively investigate the functional implications of conformational flexibility in these proteins, we have assembled a comprehensive dataset of RNA- and DNA-binding protein sequences and structures.

**Fig 1:**
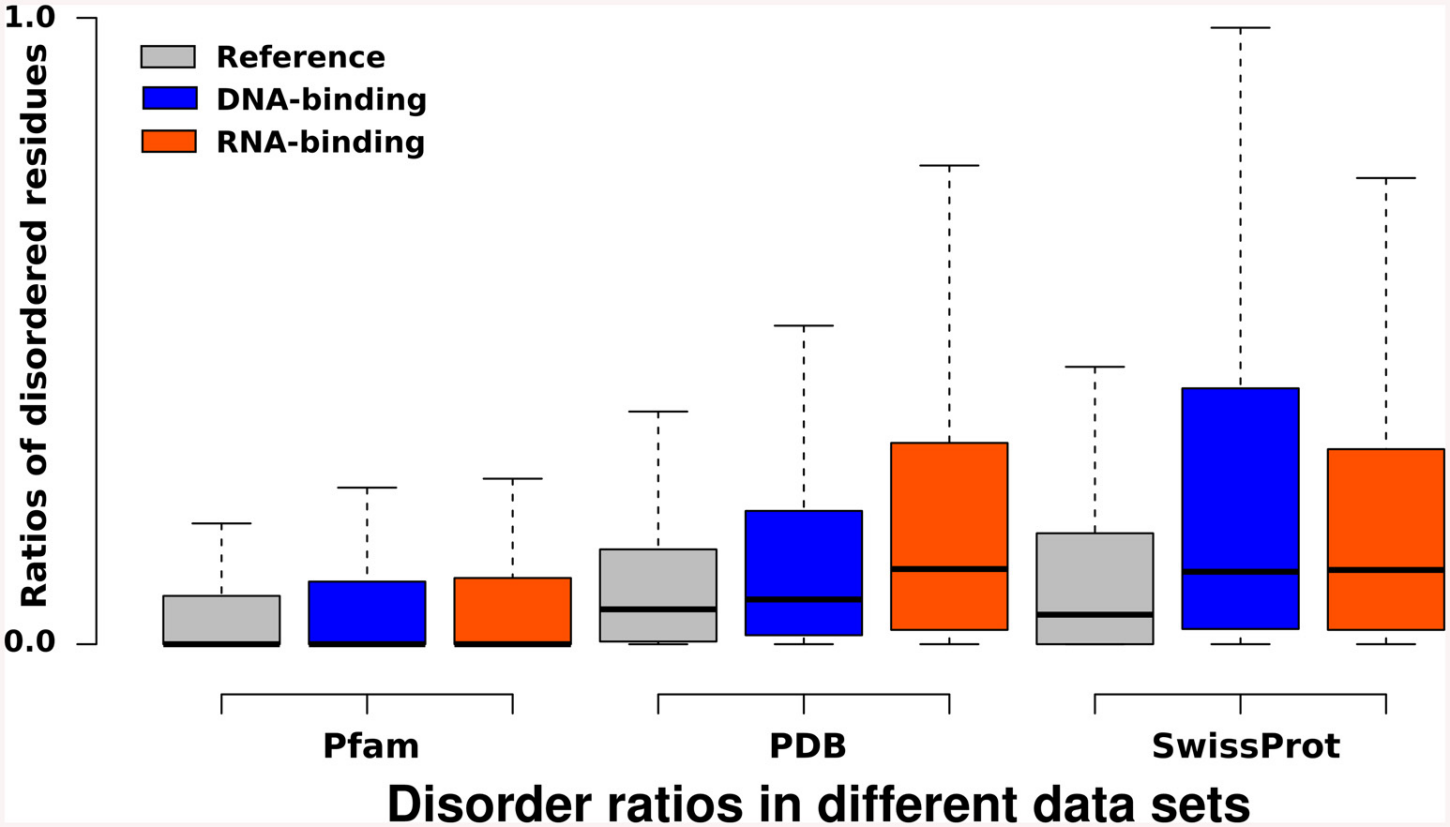
Fractions of disordered residues. Box plot of the ratios of disordered residues across three datasets: The Pfam dataset, the PDB dataset and the SwissProt dataset. DNA- (blue) and RNA- (orange) binding proteins/domains are compared to the reference datasets (grey). In all three datasets the RNA-binding proteins/domains have significantly higher disorder content than the reference data.

The initial step of the large-scale computational investigation was to calculate residue-wise disorder scores for each protein sequence in our complete dataset. One of the meaningful descriptors of disorder that can be derived from such scores is the fraction of disordered residues, which provides information on the overall disorder content of a protein chain. The distribution of the disorder ratios for the sequences across the three datasets is displayed on Fig 1.

**Fig 2:**
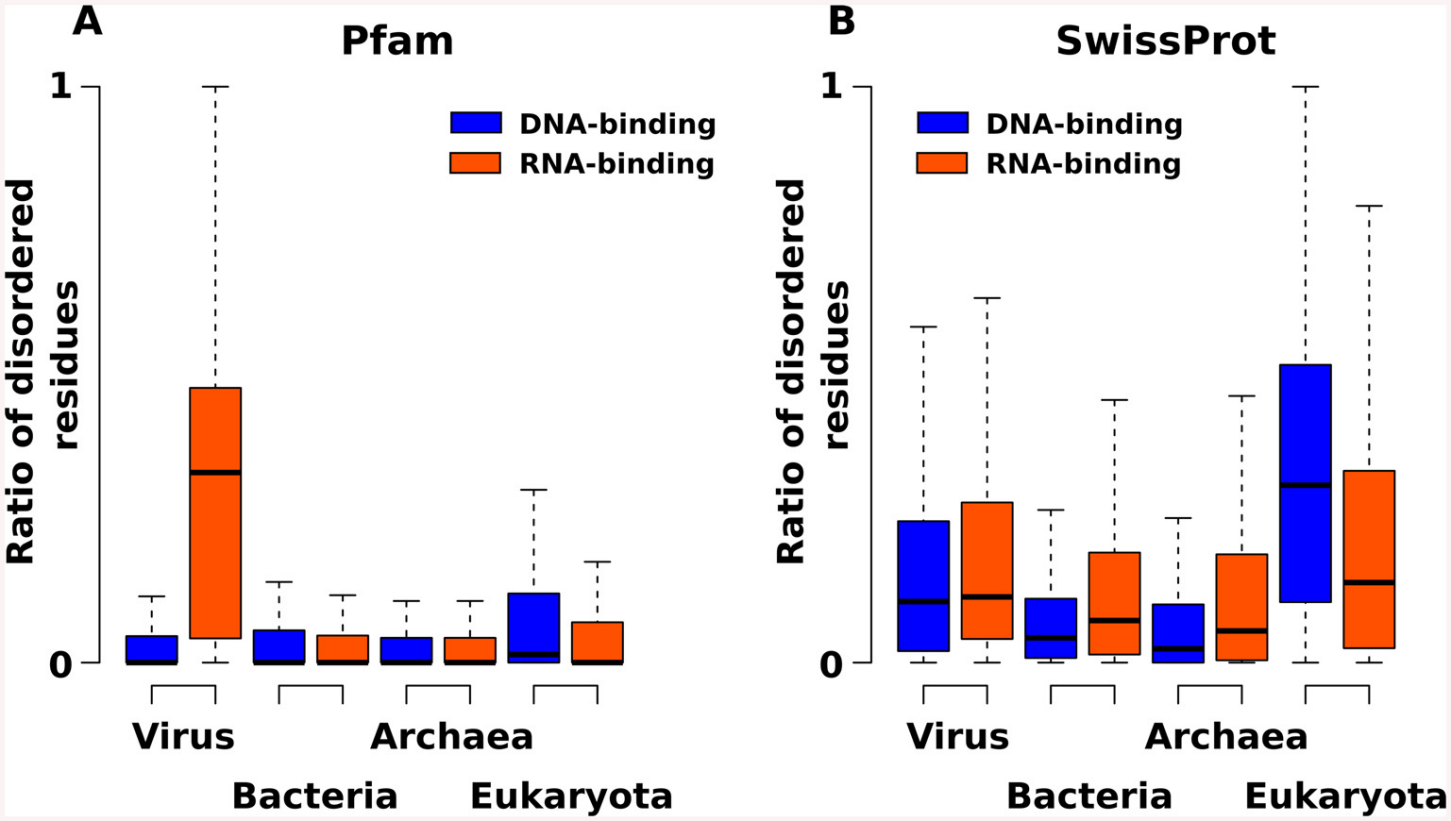
Disorder content across taxonomic groups. The two box plots compare the ratios of disordered residues across major taxonomic groups. The Pfam dataset (A) is significantly biased by viral domains which have an outstandingly high, 30% disorder content. The SwissProt dataset (B) is more balanced, where viral (19%) and eukaryotic (21%) RNA-binding proteins have the highest fraction of disordered residues, along with DNA-binding eukaryotic proteins.

In all three datasets, RNA- and DNA-binding proteins have significantly higher disorder contents than the reference datasets, as tested by two-sample Welch t-tests, yielding p-values lower than 2.2e-16. The domain sequences in the Pfam dataset have generally lower disorder ratios, compared to the PDB and SwissProt datasets. This is to be expected, since Pfam is hosting sequences of domains that most often have well-defined structures. However, RNA-binding regions are often found outside the boundaries of Pfam domains, and in fact most of the recently discovered RNA-binding sites are within such intrinsically disordered regions[24, 25]. The PDB dataset contains relatively more disorder, as flexible segments can undergo induced folding upon binding or certain conformations might be selected from the dynamic ensemble (i.e. conformational selection). The highest proportion of disordered residues is observed in the dataset of the full length RBP protein sequences of the SwissProt dataset. Upon comparing RNA binding domains (RBDs)across taxonomic groups (viruses, bacteria, archaea and eukaryota), the Pfam and SwissProt datasets show distinct differences (Fig 2).

Viral RBDs of the Pfam dataset (Fig 2A) have surprisingly high fractions of disordered residues: almost 30% of all the amino acids are predicted to be disordered, compared to bacteria (5%) and eukaryota (8%). The SwissProt dataset (Fig 2B) is more balanced, with viral RNA-binding proteins having 20% disorder, while eukaryotic RNA-binding proteins 21% on average. When compared to DNA-binding domains/proteins, RNA-binders generally have higher disorder content, except for the known eukaryotic protein sequences, where the average ratio of disordered residues in DNA-binding proteins is significantly higher (33%).

Structural disorder is generally rather abundant in eukaryotic organisms; in fact, up to 30% of the eukaryotic proteins are predicted to have intrinsically disordered regions [26]. It has been speculated, that disorder as a feature might be required for the coordination of signalling and regulatory pathways in the complex eukaryotic cells [27]. Viruses on the other hand might be enriched in disorder, because disordered regions are thought to face reduced selective pressure[28], enabling the rapid evolution of viral sequences, leading to enhanced adaptability. Additionally, viral genomes are selected to be compact, and in this regard disordered segments with a high density of functional motifs have an obvious advantage [29]. However, the unexpectedly high ratio of disorder in viral domains of Pfam could also indicate that viral proteins have less well-defined domains, and the boundary between short domains and (disordered) binding motifs is blurry. Thus we have seen that RNA-binding proteins are enriched in disorder, but is this conformational flexibility functional?

**Fig 3:**
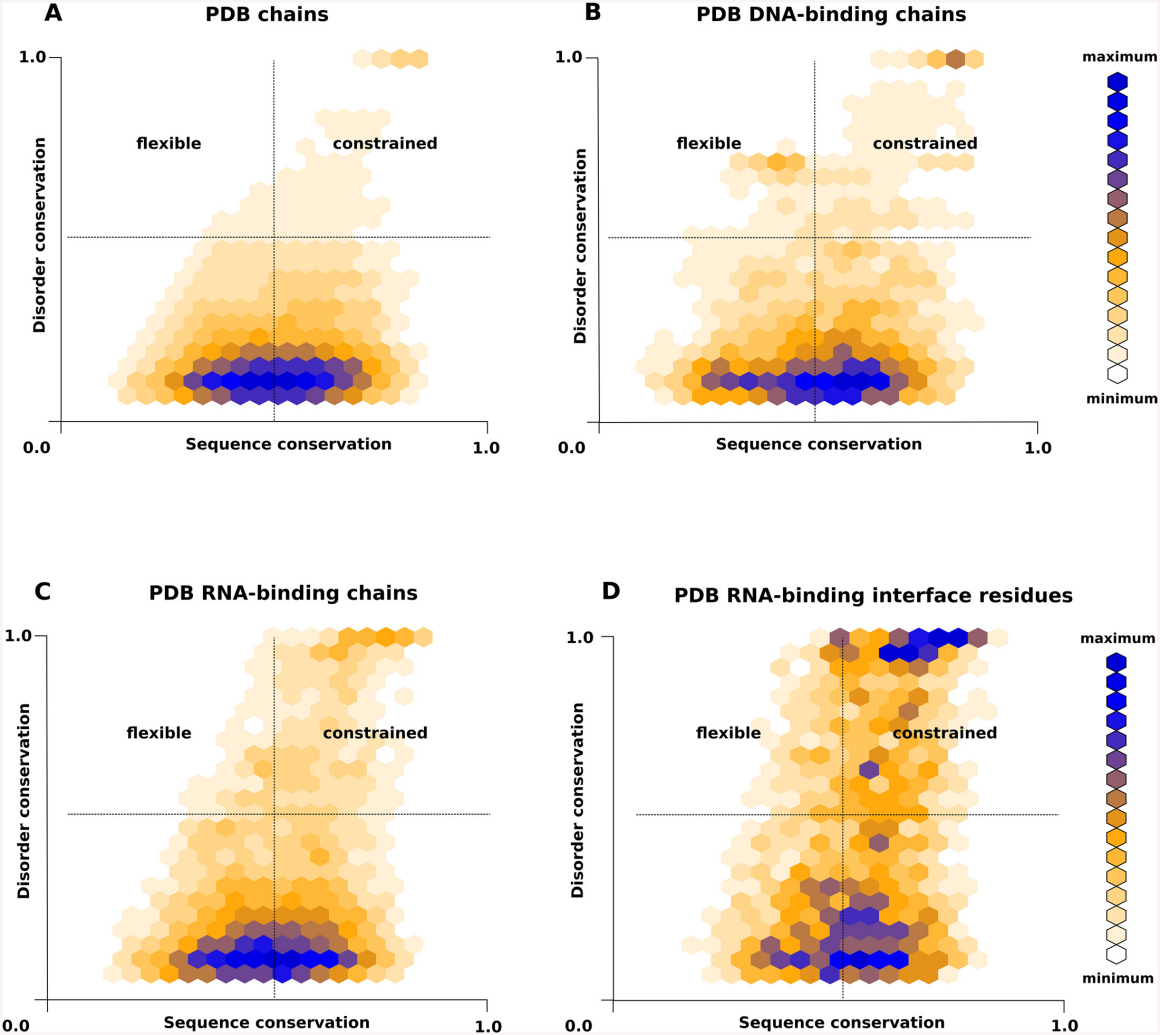
Conservation of sequence and of disorder. Heat maps of the sequence- and disorder conservation score pairs of each residue in different sets of structures. Each DisCons [13] score pair corresponds to a specific position in a multiple sequence alignment. The score pairs are binned, and the bins are colour coded: from light orange (few) to dark blue (many). Disorder is more conserved in RNA-binding protein chain (C) and especially in the RNA-binding interface residues (D) than in protein- or DNA-binding protein chains (A and B respectively).

The conservation of an important feature, such as the amino acid sequence of a protein, or the presence of intrinsic disorder may help identify functionally important protein segments. We have used our recently developed disorder conservation analysis pipeline, DisCons[13], to quantify the conservation of sequence and of disorder in an evolutionary context. Following the nomenclature of Bellay et al.[14], DisCons classifies disordered positions into three relevant categories: i.) 'Constrained', if both disorder propensity and amino acid sequence are conserved; ii.) 'Flexible', if the sequence shows high degree of variability, yet disorder as a feature is conserved; and finally iii.) 'non-conserved', if the disorder of a position is not consistent. First, we analysed the PDB dataset to provide a background against which to quantify the conservation of disorder in RNA-, DNA- and protein-binding proteins. Disorder and sequence conservation score pairs of each position in the PDB dataset are displayed on Fig 3.

As shown by two-sample Kolmogorov-Smirnov tests, disorder in RNA-binding proteins is significantly more conserved (p-value less than 2.2e-16), than in DNA-binding proteins, or proteins of PDB in general. Additionally, disorder in RBPs is often conserved even when the underlying amino acid sequence is not, indicating the functional importance of the lack of structure, rather than of specific residues within such disordered regions. The conservation of disorder is even more pronounced for residues that are in direct contact with RNA, i.e. RNA-binding interface residues (Fig 3D). On the binding interfaces 'constrained' disorder dominates, as both sequence and structural disorder are strongly conserved.

**Fig 4:**
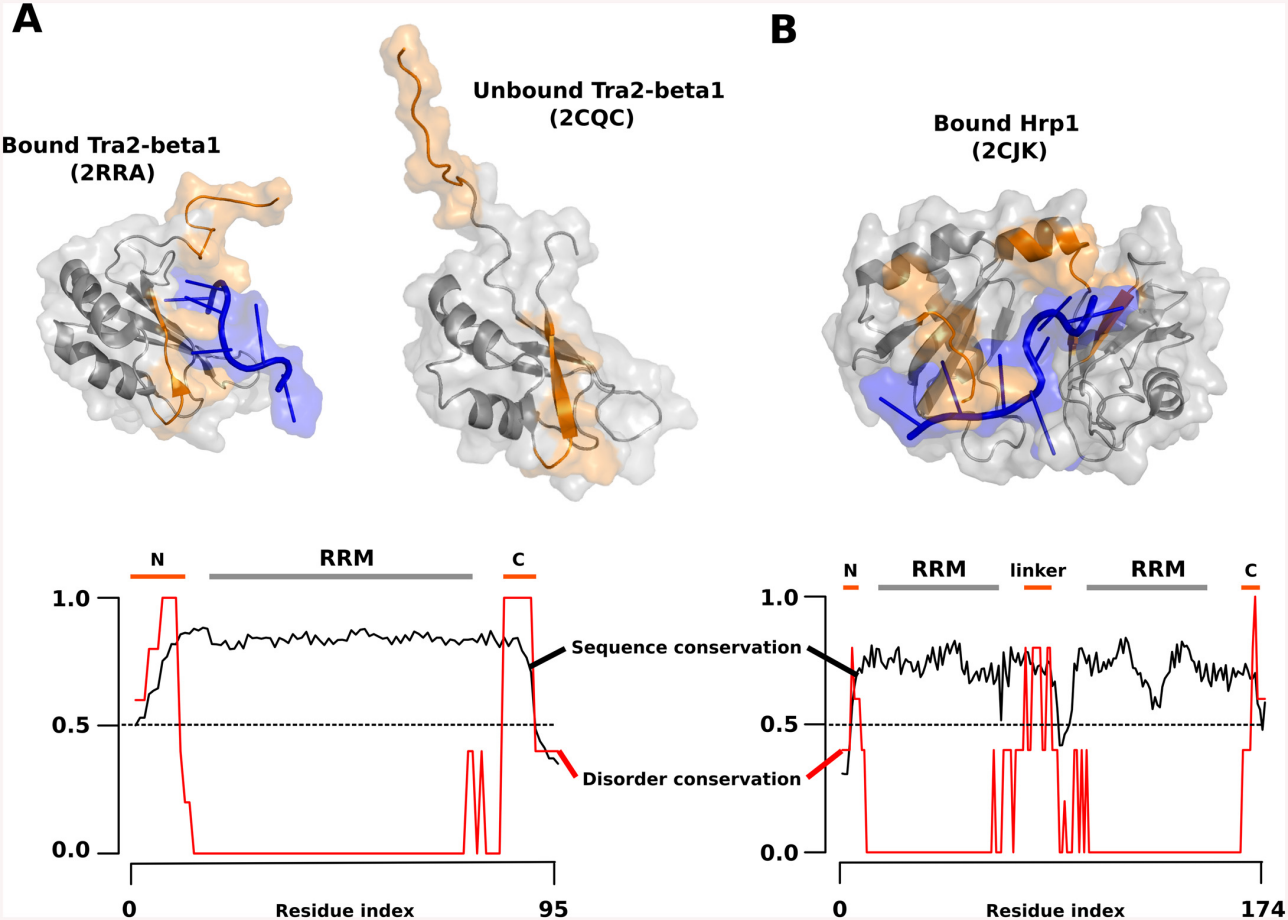
Examples of constrained disorder. Two examples of 'constrained' disorder, where both the sequence and the disorder feature are conserved. In the RRM domain of splicing factor Tra2-β1 in complex with RNA (A) both the N- and C-termini regions (orange)adopt a folded state and form extensive interface contacts (left side of panel A) with RNA (blue), whereas they are flexible in the free form (right side of panel A). In the case of Hrp1 protein (B) RNA recognition and binding occurs via tandem RRM modules, and the termini along with the inter-domain linker (orange)are also implicated as a key player in the interaction. This linker is flexible in the unbound state, and forms a short alpha-helix when in complex with RNA (blue). At the bottom of panel A and B the respective sequence and disorder conservation profiles are shown.

Fig 4 provides two specific examples of 'constrained' disordered interfaces in protein-RNA complexes. The protein chain segments that border the members of the RNA Recognition Motif (RRM) domain family often also play important roles in RNA-binding. In many known examples these flanking segments undergo disorder-to-order transitions that juxtapose them with RNA. The additional contacts serve to increase the total interaction surface, thus they enhance the binding affinity and also tune specificity of the interaction.

One such example is the RRM domain of splicing factor Tra2-β1 in complex with RNA (PDB IDs 2CQC, bound; and 2RRA, unbound) (Fig 4A). Here, both the N- and C-terminal regions of the RRM are disordered in the free state, but adopt a folded structure in the complex with RNA, forming extensive contacts [30]. Therefore, the complete interface consists not only of the canonical β-sheet residues of the RRM, but also encompasses the terminal residues flanking the RRM. Interaction with the N- and C-terminal extensions not only increases binding affinity, but it is also important for specificity, as recognition of the RNA involves hydrogen bonding with several of the RRM flanking residues[31]. It is very likely that the structural ordering of the two termini of the RRM in the complex serves yet another important purpose. The build-up of Tra2-β1 is unique in the sense that its RRM is located between two RS (arginine-serine) domains, and the folding of the RRM-flanking linkers upon RNA binding also induces the correct positioning of the RS domains. Furthermore, the folding of the disordered termini might also assist in the formation of functional protein-protein interactions of human Tra2-β with other splicing factors. Direct interactions of human Tra2-β with two such novel splicing factors, hnRNP-G and SRp30c modulate the inclusion of exon7 of the survival motor neuron gene (SMN2) in the final transcript, preventing the lethal condition of proximal spinal muscular atrophy (SMA) [32, 33]. In accordance with the functional importance of the terminal residues of the RRM in Tra2-β1, our analysis of sequence and disorder conservation (Fig 4A, bottom) indicates that both the N- and C-terminus of this domain are segments of 'constrained' disorder, underlining that the functional importance of their interactions with RNA and additional protein partners are manifested in the conservation of both their sequence and disorder. The sequence of the RRM itself is highly conserved, and in addition the sequences of the flanking segments are also strongly conserved. Disorder within RRM, a well-structured fold, is low; the flanking regions, however, are disordered and, importantly, this disordered nature is consistent across all the aligned sequences.

Another biologically relevant example of the role of disordered N- and C-terminal extensions of RRMs for RNA recognition is provided by the conservation of sequence and disorder in the case of mRNA 3’UTR recognition by the nuclear polyadenylated RNA-binding protein 4 (Hrp1) (Fig 4B). In this instance, RNA recognition and binding occurs via tandem RRM modules. In addition to the primary RNA binding surfaces offered by the beta-sheets of both RRMs and their C- and N-terminal flexible residues, the inter-domain linker (connecting RRMs 1 and 2) also plays a critical role in the interaction. While the linker forms a short alpha-helix in the crystal structure of the protein-RNA complex (PDB 2CJK, bound) [34], it is disordered in the unbound state and by NMR chemical shift differences it undergoes significant structural changes. The helix contains a large number of charged residues which make it disordered in the isolated form, and are important in stabilizing the complex with RNA through salt-bridge interactions. In accordance with its importance in binding to the RNA, the linker region is predicted to be of 'constrained' disorder (Fig 4B). This mode of RNA recognition, which involves active participation of the linker, is also seen in the crystal structures of Sex-lethal [35], PABP [36], HuD [37] and nucleolin[38]: in all these cases, the linker connecting the two RRM domains is disordered in the free protein, and becomes folded in the complex with RNA.

### Structural Consequences of Conformational Flexibility

**Fig 5:**
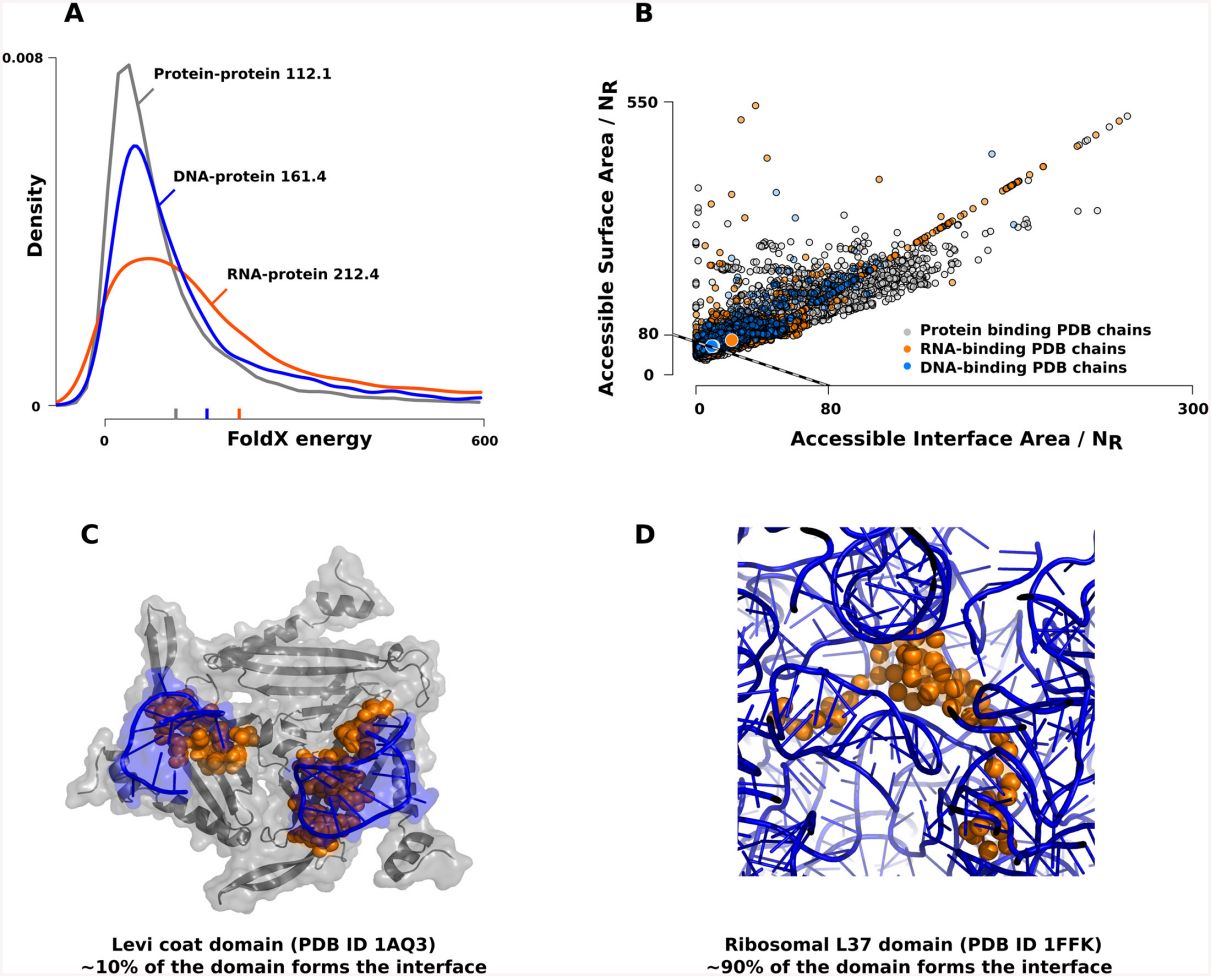
Consequences of disorder on conformational energies and interface area. According to energy calculations with FoldX (A), RNA-binding protein chains are significantly less stable than DNA-binding and protein-binding protein chains in the PDB database, indicating that the unbound conformations are either flexible or conformationally different than in the bound forms. On the other hand, the relative size of the binding interfaces tend to be significantly higher in RNA-binding proteins (B). The smaller circles on the plot are individual chains, while the large circles are the average for each of the following groups: ‘gray’ for protein-binding chains, ‘orange’ for RNA-binding chains and ‘blue’ for DNA-binding chains. On average, RNA-binding chains are the most likely to be flexible in the unbound conformation. Two examples from the spectrum of relative interface sizes and disorder content are shown on panels C and D.

We predicted that a significant fraction of the residues in RNA-binding proteins are intrinsically disordered, and showed that their disorder is evolutionarily conserved, especially in the regions that constitute the binding interfaces. Such a strong enrichment of flexible residues should have significant effects on the conformational stability of these proteins. To test this, we calculated the conformational energies of each protein chain in our PDB dataset using the energy scoring function of FoldX [20](Fig 5A). According to two-sample Welch t-tests, the structures of RNA-binding chains are significantly less stable (higher energies), in comparison to DNA-binding or protein-binding protein chains. This indicates that most of the RNA-bound protein structures found in PDB are likely to be unstable in the unbound form, and are only stabilized by binding to RNA. Indeed, when the normalized accessible surface area and the normalized interface area of each chain are displayed (Fig 5B), RNA-binding protein chains often occupy the area of the plot that is specific to disordered proteins that fold upon binding according to Nussinov et al[21], forming relatively large interaction interfaces. It appears that RNA-binding proteins are more disordered, and make larger interfaces than DNA- and protein-binding proteins. In fact, there is a positive correlation (Pearson correlation coefficient of 0.44) between the ratio of disordered residues and the normalized size of interaction interfaces in this class of proteins. Fig 5C and 5D provide examples of this correlation. The formation of the interface in the protein-RNA complex of the Levi coat domain (PDB ID 1AQ3) includes approximately 10% of the domain residues, and has a disorder content of 24% (Fig 5C). In contrast, nearly 90% of the residues are in interaction with RNA in the complex of the ribosomal L37 domain, which has a disorder content of 43% (Fig 5D).

Even though most of the disordered regions of RNA-binding proteins appear to obtain a more rigid conformation upon binding, only a fraction of the regions adopt regular secondary structural elements. Overall, around a quarter of every disordered residues adopts either helical (16.2%) or strand (11.1%) conformations. This ratio is even smaller on the binding interfaces, where only every fifth residue occurs within regular secondary structural elements. This indicates that while the global conformation of the RNA-binding proteins tends to be compact, the disordered regions generally remain more extended even in the bound form, and often wrap around RNA-segments, making large interfaces.

### Functional Implications of Intrinsic Disorder

**Fig 6:**
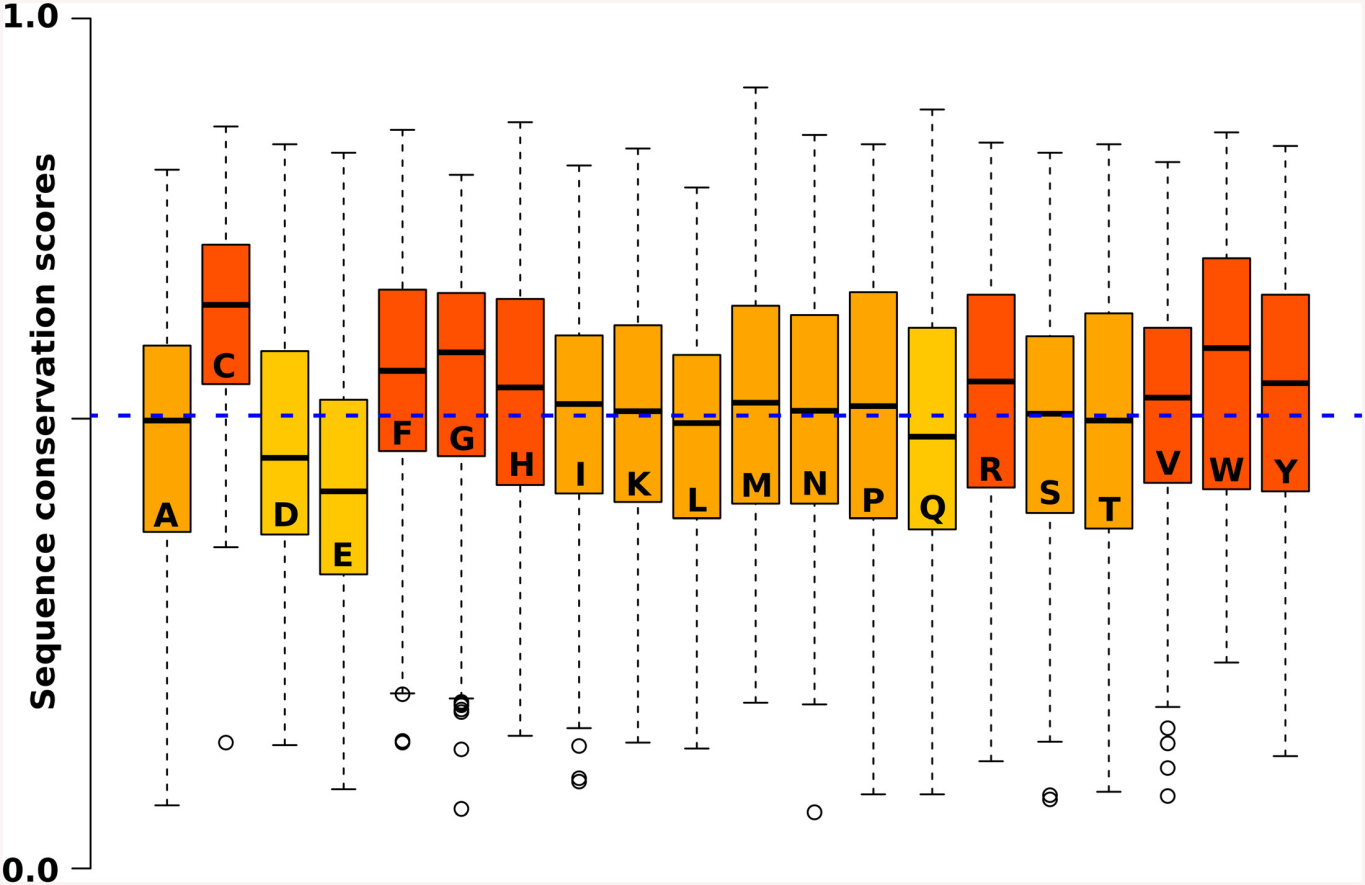
Amino acid specific conservation scores on disordered regions. Position-specific conservation score for each amino acid across the disordered regions of RNA-binding proteins. Negatively charged residues are less conserved, while arginines are more conserved than the average (blue dashed line). However, additional residues are also significantly more conserved than the average. Residues with mean sequence conservation scores significantly higher than that of the overall dataset are darker orange, while significantly less conserved residues are lighter orange.

Disordered regions are enriched in disorder-promoting amino acids, such as glycine, proline or arginine, and are depleted in order-promoting hydrophobic residues that could form a stable hydrophobic core [39]. Ribosomal proteins are known to be enriched in positively charged residues [40], and this feature is generally true for RNA-binding proteins. Compared to the background amino acid composition of the complete PDB dataset, we found that there is a 40% increase in the relative amount of arginines and a 33% increase in lysines. When considering only those residues that are in direct contact with RNA, these numbers further increase tremendously: 180% in the relative amount of arginines and 116% in lysines. Such significant biases in the amino acid composition clearly indicate the importance of electrostatic interactions in RNA-binding, and could also account for the prevalence of intrinsic disorder. Upon examining the sequence conservation of each residue type on the RNA-binding interfaces, arginines were found to be slightly more conserved than the average, while the most conserved residues in disordered regions were cysteins, glycines and tryptophanes (Fig 6). This conservation pattern indicates the functional importance of those residues that do not actively participate in establishing electrostatic interactions, but may offer additional features, such as unusual aspects of the polypeptide backbone or hydrophobic interactions with bases of the nucleotides. A further benefit may be provided by ‘fly-casting’, i.e. a binding rate acceleration as a result of a relatively large capture radius of IDPs compared to structured protein segments[41].

Besides supporting a high number of favourable electrostatic interactions and increasing the capture radius of the protein, there is yet another major advantage of conformational flexibility, which is its multi-functionality or ‘moonlighting’ [42]. It has been shown that arginine-rich RNA-binding motifs (ARMs) may bind, different RNAs by adapting to different binding surfaces[43, 44]. One such classical example is the Tat protein of the Jembrana disease virus (JDV), which can bind different TAR RNA sites [43]. These proteins are unique transcription factors, which bind mRNA transcripts rather than DNA. The RNA-recognition site of JDV Tat was found to bind not only to its native TAR site, but also HIV and BIV TAR RNAs. The conformation of the bound ARM of Tat is context dependent and unique in each interaction. It has been speculated that while arginines play a key role in establishing electrostatic interactions, the other residues are responsible for providing specificity via negative steric and electrostatic effects [44], which could explain the conservation of additional amino acids found in IDRs of RNA-binding proteins.

**Fig 7:**
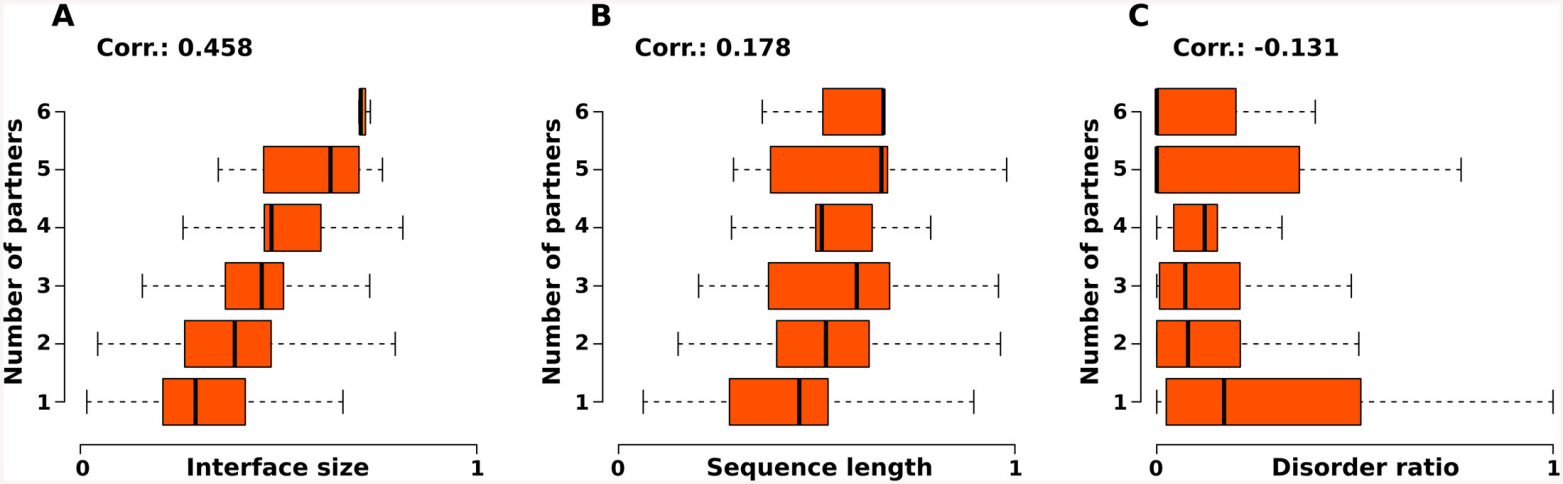
Parameters that are correlated with the number of interacting partners. In order to test the hypothesis that disorder is favourable for allowing the protein chains to act as molecular scaffolds, we investigated several parameters in correlation with the number of bound partners in RNA-protein complexes. While the number of partners is positively correlated with the area of the interaction interface (A), and slightly with the length of the sequence, it is weakly and negatively correlated with the ratio of disordered residues. All three parameters were normalized.

Additionally, conformational flexibility could be favourable in allowing a multi-domain protein chain to act as a scaffold by binding multiple protein and nucleic acid partners at the same time. In order to test this hypothesis, we investigated the correlation of distinct features of the RNA-binding chains with the number of their partners, with focus on the possible effects of intrinsic disorder (Fig 7). Apparently, the number of partners does not increase with higher flexibility of the protein chain; on the contrary, there is a slight negative correlation (Kendall's tau -0.131). Of the examined parameters, only the area of the interface (Pearson 0.458) and the length of the sequence (Kendall's tau 0.178) show positive correlation with the number of bound partners in the complex. Based on these findings it seems unlikely that the increase of disorder content in RNA-binding proteins was driven by an optimization towards functioning as molecular scaffolds.

## Discussion

RNA-binding proteins have often been associated with intrinsic disorder, yet the functional advantages of flexibility remain unclear [5]. Intrinsically disordered regions that make contacts with protein or nucleic acid partners undergo induced folding or disorder-to-order transitions to make a more structured conformational state[6]. Such transitions have an inherent entropic cost, which may make the interaction weaker than between rigid partners[5]. In this work, we provide a comprehensive and detailed overview on the prevalence and role of structural disorder RNA-binding proteins.

We show that RNA-binding proteins are significantly enriched in disorder, and that a major fraction of the disordered residues are found within the binding interfaces that are in direct contact with RNA. Disordered interface residues tend to fall into the category 'Constrained' disorder, since both their amino acid sequence and their disorder feature are highly conserved. In contrast, in the full length RNA-binding protein chains, the conservation of disorder is twice as high, as the conservation of the underlying sequence. In this latter case the function of the protein segment relies more upon the overall structural flexibility than on particular amino acid residues, which is in line with the general idea that disordered regions face less stringent evolutionary pressure [45], as for example demonstrated by the HIV Rev motif. Rev is an ARM, much like Tat, and it has been shown that this disordered segment is robust against substitutions[46]. Based on the distinct differences in the conservation profiles of *de facto* binding residues and those that mainly function as flexible linkers, it would seem likely that tools, such as DisCons[13]that investigate and quantify the conservation of both the amino acid sequence and the disordered nature of a protein may offer an additional layer of information that can complement and enhance the performance of RNA-binding site prediction software, such as RNABindR [47], NAPS[48] or RNAProB[49], even though some of these methods already take evolutionary information into account. While the accuracy of these tools is progressively getting higher, it was shown recently that different methods sometimes yield conflicting predictions [50]. Evaluating the conservation profiles in case of such conflicts could serve as cross-validation and may provide additional support for the validity of a specific prediction.

The disordered and conserved residues that interact with RNA are significantly biased in their amino acid composition, having more than twice as much positively charged residues as the average in PDB. These disordered and positively charged chains form extended segments that maximize the interfaces between RNA and protein, supporting the notion that disordered regions are able to establish well-fitted and larger interaction interfaces than their folded counterparts[51, 52]. Additionally, the stability of the structure of RNA-binding protein chains is affected drastically by the abundance of intrinsically disordered residues.

All together, these findings demonstrate that the main functional contribution of intrinsic structural disorder in RNA-binding proteins is that it allows the formation of large, extended interaction interfaces dominated by electrostatic interactions. Another important role of conformational flexibility is to support multi-functional regions, such as the ARM sites, which can target different RNA partners via context dependent binding-induced folding[7, 43]. Such multi-functionality is especially favourable for viruses, in which new functional protein-RNA interactions may evolve rapidly, without non-functional intermediates [44], coupled with the additional advantage of genome compaction[29].

## Conclusions

In this study we presented a comprehensive analysis on the enrichment of structural disorder in RNA-binding proteins, and look for possible explanations of this phenomenon in terms of the functioning of IDRs through disorder-to-order transitions. Since such conformational changes are entropically expensive, the functional advantages of excessive conformational flexibility is questionable. We suggest that intrinsic disorder provides for two major advantages: First, these proteins establish large, extended electrostatic interaction interfaces dominated by positively charged, conserved disorder-promoting residues. Tight contacts within these large interfaces is a result of induced fit [52], which in combination with the ‘fly-casting’ effect can accelerate and optimize molecular recognition. Second, conformational flexibility makes multi-functionality (i.e. ‘moonlighting’) feasible by targeting different RNA partners with the same disordered protein segment, by acquiring conformations in a context-dependent manner[7, 43]. While positively charged residues within these IDRs contribute to electrostatic interactions, other residues provide specificity, mostly by negative steric effects. Such multi-functionality also supports genome compaction and the rapid evolution of new interactions without the disadvantage of non-functional intermediates.
